# Gamma Interferon Is Required for Optimal Antibody-Mediated Immunity against Genital Chlamydia Infection

**DOI:** 10.1128/IAI.00749-16

**Published:** 2016-10-17

**Authors:** Elizabeth K. Naglak, Sandra G. Morrison, Richard P. Morrison

**Affiliations:** Department of Microbiology and Immunology, University of Arkansas for Medical Sciences, Little Rock, Arkansas, USA; Washington State University

## Abstract

Defining the mechanisms of immunity conferred by the combination of antibody and CD4^+^ T cells is fundamental to designing an efficacious chlamydial vaccine. Using the Chlamydia muridarum genital infection model of mice, which replicates many features of human C. trachomatis infection and avoids the characteristic low virulence of C. trachomatis in the mouse, we previously demonstrated a significant role for antibody in immunity to chlamydial infection. We found that antibody alone was not protective. Instead, protection appeared to be conferred through an undefined antibody-cell interaction. Using gene knockout mice and *in vivo* cellular depletion methods, our data suggest that antibody-mediated protection is dependent on the activation of an effector cell population in genital tract tissues by CD4^+^ T cells. Furthermore, the CD4^+^ T cell-secreted cytokine gamma interferon (IFN-γ) was found to be a key component of the protective antibody response. The protective function of IFN-γ was not related to the immunoglobulin class or to the magnitude of the Chlamydia-specific antibody response or to recruitment of an effector cell population to genital tract tissue. Rather, IFN-γ appears to be necessary for activation of the effector cell population that functions in antibody-mediated chlamydial immunity. Our results confirm the central role of antibody in immunity to chlamydia reinfection and demonstrate a key function for IFN-γ in antibody-mediated protection.

## INTRODUCTION

Chlamydia trachomatis is an obligate intracellular bacterium responsible for more than 1.4 million genital infections annually in the United States and for approximately 106 million genital infections annually worldwide (1). Although C. trachomatis infection is the most common bacterial sexually transmitted infection (STI), there is currently no vaccine to aid in disease prevention. Genital infections in women are typically asymptomatic and can lead to severe pathology, including pelvic inflammatory disease (PID) and tubal factor infertility. With a great need for the development of an efficacious vaccine, it is important to understand the natural protective immunity that arises following genital infection with Chlamydia. The C. muridarum model of murine genital tract infection has proven very useful for such studies as this model closely recapitulates the disease state observed in women, including ascension of Chlamydia from the lower to the upper genital epithelium and irreversible pathological sequelae.

Adaptive immune responses are required to resolve C. muridarum genital infection and to protect against reinfection. Athymic mice, major histocompatibility class (MHC) II knockout mice, T cell receptor (TCR) αβ knockout mice, and mice depleted of CD4^+^ T cells are unable to resolve primary genital chlamydia infection (2–5). Among the cellular responses, CD4^+^ T cells appear to be primary effectors in the resolution of genital infection. While the mechanisms for CD4^+^ T cell-mediated protection are not fully defined (6), some evidence has suggested that immunity provided by CD4^+^ T cells occurs through Plac8-mediated T cell degranulation (7) and gamma interferon (IFN-γ) production (4, 8–10).

The humoral arm of the adaptive immune response also contributes very importantly to chlamydial immunity. Antibody speeds the resolution of primary infection, provides a level of protection to reinfection that is equivalent to that of CD4^+^ T cells (5, 11, 12), and has been implicated in a protective role in vaccine studies (13, 14). Interestingly, CD4^+^ T cells appear to be playing a dual role in the protective response against C. muridarum, as they not only function to resolve primary infection independently of CD8^+^ T cells and antibody (12) but also function cooperatively with antibody (5, 6, 12). Several lines of evidence support the idea of a cooperative function between antibody and CD4^+^ T cells and provide evidence that CD4^+^ T cells are responsible for the recruitment and/or activation of a local effector cell population that functions with antibody to resolve infection. For example, the passive transfer of convalescent (immune) serum to naive C57BL/6 mice depleted of CD4^+^ T cells fails to protect the mice against primary Chlamydia genital infection (mice are unable to resolve infection and shed very high numbers of bacteria). However, administration of that same serum to CD4^+^ T cell-sufficient naive mice at the time of primary infection decreases the infectious burden and shortens the course of infection compared to mice that do not receive immune serum (12). The protection afforded to naive mice by immune serum is particularly notable at days 10 to 14 of the infection, a time when the numbers of CD4^+^ T cells in the genital tract tissue are increasing (12). Lastly, antibody imparts a striking level of protective immunity against reinfection in the absence of CD4^+^ T cells (11). Collectively, these results strongly support the notion that the protective mechanism for antibody in chlamydial immunity is not direct bacterial neutralization (blocking of attachment) *per se* but that protection is instead mediated through the interaction of antibody with a yet-to-be-identified effector cell population.

Knowing that antibody alone does not protect a naive genital tract from chlamydial infection and is protective only following CD4^+^ T cell priming (11, 12), we reasoned that CD4^+^ T cell priming of the genital tract led to the recruitment and/or activation of an immune effector cell that interacts with antibody to confer protection. CD4^+^ T cells are known to secrete a variety of proinflammatory cytokines and chemokines that are capable of recruiting and activating other immune cells at the site of infection (4). One such cytokine that is produced in response to chlamydial genital infection is IFN-γ (4). Because IFN-γ is produced by CD4^+^ T cells and is known to activate cell populations that interact with antibody (e.g., macrophages) to kill pathogens, we sought to investigate the role of IFN-γ in antibody-mediated immunity to genital chlamydia.

In this study, we showed that the CD4^+^ T cell-secreted IFN-γ cytokine was necessary for optimal antibody-mediated immunity. The impact of IFN-γ on the protective response did not appear to be due to the recruitment of an effector cell population or to a change in the antibody response. Rather, our data support the hypothesis that CD4^+^ T cells cooperate with antibody in providing a striking level of protective immunity through the activation of an effector population that interacts with antibody to eliminate infectious chlamydiae.

## MATERIALS AND METHODS

### Mice.

Female, 6-to-8-week-old wild-type C57BL/6, μMT (antibody-deficient) (B6.129S2-*Igh-6*^*tm1/Cgn*^/J), and IFN-γ^−/−^ (B6.129S7-*IFN-g*^*tm/1Ts*^/J) mice were purchased from Jackson Laboratory and housed in the animal facilities at the University of Arkansas for Medical Sciences (Little Rock, AR). Animal care and use protocols were approved by Institutional Animal Care and Use Committee and followed institutional guidelines.

### Chlamydia growth and purification.

C. muridarum (Weiss strain) was propagated in HeLa 229 cells, and infectious elementary bodies were harvested by density gradient purification as previously described (15).

### Genital infection and quantitation of chlamydiae.

Mice were injected subcutaneously with 2.5 mg of medroxyprogesterone acetate (Depo-provera) (Greenstone LLC) 10 and 3 days prior to primary infection and 5 days prior to secondary infection to synchronize the estrous cycles of the experimental mice. Prior to infection, the vaginal vault was gently swabbed with a Calgiswab (Puritan) to remove any mucus plugs. Infection was established by placing 5 μl of a mixture of 250 mM sucrose, 10 mM sodium phosphate, and 5 mM l-glutamic acid (pH 7.2) (SPG) containing 5 × 10^4^ inclusion-forming units (IFUs) (100 50% infective doses [ID_50_]) into the vaginal vault. Reinfection inoculations occurred 66 days after the onset of primary infection.

To assess bacterial infection, cervicovaginal swabs were collected on days 3, 7, 10, and 14 and then every 7 days postinfection until infection resolved. Swabs were placed in 2-ml tubes with 0.5 ml SPG and 2 sterile 4-mm-diameter borosilicate glass beads (Fisher). Tubes were subjected to vortex mixing for 2 min at 4°C at 1,400 rpm using a Thermomixer R incubator (Eppendorf). Swabs were removed, an additional 0.5 ml of SPG was added to each tube to reach a total volume of 1 ml, and samples were stored at −80°C. Bacterial load was assessed by enumeration of IFUs on HeLa 229 monolayers (3). HeLa 229 cells were seeded at 2 × 10^5^ cells/well in 48-well cell culture plates. The following day, monolayers were washed with Hanks' balanced salt solution (HBSS) and treated with HBSS containing 45 μg/ml DEAE dextran for 10 min at room temperature. Cells were washed once with HBSS and infected with 300 μl of vaginal swab collections. Plates were centrifuged for 1 h at 37°C and 800 × *g* and then rested at 37°C for 30 min. Monolayers were then washed once with HBSS to remove unattached chlamydiae, and 0.5 ml Dulbecco's modified Eagle medium containing 10% fetal bovine serum (DMEM-10), 10 μg/ml gentamicin (Invitrogen), and 1 μg/ml cycloheximide was added to each well. Monolayers were incubated at 37°C in a humidified atmosphere containing 5% CO_2_ for 20 to 24 h. Following incubation, the medium was removed and monolayers were fixed with methanol for 10 min and washed once with HBSS. IFUs were detected using an anti-major outer membrane protein (MOMP) monoclonal antibody (Mo33b) followed by goat anti-mouse fluorescein isothiocyanate (FITC) antibody (MP Biomedicals) secondary staining.

### CD4^+^ T cell depletion.

CD4^+^ T cells were depleted *in vivo* by injecting anti-CD4 (clone GK1.5) intraperitoneally as described previously (11). Briefly, mice received 0.5 mg of anti-CD4 on days 6, 5, and 4 prior to primary or secondary infection, followed by 0.3 mg of anti-CD4 beginning on day −1 and continuing every 3 days through day 20 postinfection. This depletion regimen depletes >99% of CD4^+^ T cells in the spleen and genital tract tissue, and CD4^+^ T cells remain depleted for approximately 7 days after the last anti-CD4 injection (5).

### Antibiotic treatment.

Prior to secondary infection, all mice received 0.1 ml (3 mg/ml) doxycycline hyclate (MP Biomedicals)–distilled water intraperitoneally beginning on day 42 post-primary infection to ensure infection resolution. Injections were given daily for a period of 2 weeks, through day 55. Mice were then rested for 10 days prior to secondary inoculation.

### Antibody collection and chlamydial ELISA.

Mice were bled on days 32, 58, and 83 post-primary infection, and serum was collected. Serial dilutions of sera were tested for anti-C. muridarum antibodies by enzyme-linked immunosorbent assay (ELISA) as previously described (3). Briefly, formalin-fixed, density gradient-purified C. muridarum elementary bodies were used as the antigen, and alkaline phosphatase-conjugated goat anti-mouse antibodies IgG1, IgG2b, IgG2c, IgG3, and IgA (Southern Biotech) were used to assess the anti-C. muridarum antibody response. ELISA titers are reported as the highest dilution of serum with an absorbance result (optical density at 405 nm [OD_405_]) of at least 0.25, which is >3 times the absorbance of 1:16-diluted serum from uninfected mice.

### Flow cytometry.

Whole murine genital tracts were harvested and minced with sterile scissors in RPMI medium (Life Technologies) with 1.5 mg/ml collagenase IV (Worthington Biochemical Company). Tissue and fluid were transferred into C-tubes (Miltenyi Biotec) and incubated rotating at 37°C for 30 min. Samples were then homogenized twice using the m_intestine_01 program on a gentleMACS Dissociator (Miltenyi Biotec) per the manufacturer's protocol. A 5-ml volume of fresh collagenase was added, and samples were incubated 20 min rotating at 37°C. Samples were again homogenized twice as noted above. Samples were then rinsed through a 70-μm-pore-size cell strainer (ThermoFisher) and centrifuged. Pellets were washed in fluorescence-activated cell sorter (FACS) buffer (1× phosphate-buffered saline [PBS]–0.1% bovine serum albumin [BSA]), and red blood cells were lysed in Tris-NH_4_Cl (170 mM Tris base, 160 mM NH_4_Cl) (pH 7.65) for 4 min at room temperature. Cells were then washed once in FACS buffer and centrifuged at 1,500 rpm at 4°C for 5 min. Cells were resuspended in 0.5 to 1 ml FACS buffer, counted using an Auto 1000 Cellometer (Nexcelom Bioscience), and adjusted to reach a volume of 1 × 10^6^ cells/ml. Mouse BD Fc Block (BD Pharmingen) was added to the suspension of genital tract cells, and genital tract cells were stained for 30 min at 4°C with the following antibodies in FACS buffer: anti-CD8-peridinin chlorophyll protein (PerCP)-eFluor 710 (53-6.7; eBioscience), anti-CD11b-Alexa Fluor 488 (M1/70; eBioscience), anti-CD45R (B220)-phycoerythrin (PE)-Cyanine7 (RA3-6B2; eBioscience), anti-NK1.1-PE (PK136; eBioscience), anti-CD11c-Alexa Fluor 700 (N418; eBioscience), anti-CD4-eFluor 660 (GK1.5; eBioscience), anti-Ly6G (Gr-1)-eFluor 450 (RB6-8C5; eBioscience), and anti-F4/80-PE-eFluor 610 (BM8; eBioscience). Following staining, cells were washed with FACS buffer and fixed in 4% paraformaldehyde prepared in 1× filter-sterilized PBS. All samples were analyzed using a BD LSRFortessa cell analyzer (BD Biosciences) and FlowJo v10.1r1. The gating strategy was determined using Fluorescence Minus One (FMO) controls (16).

### Quantitative real time-PCR (qRT-PCR) gene standards.

C57BL/6 arginase I (Arg1) and inducible nitric oxide synthase (iNOS) genes were accessed via GenBank accession no. NM_007482.3 and NM_010927.4, respectively. Based on the predicted TaqMan primer/probe assay (Applied Biosystems) locations given by the manufacturer, a fusion was synthesized with Arg1 bp 54 to 331, linked to iNOS bp 2648 to 2924. A NcoI site was added to the start of Arg1, and overlapping BamHI (GGATCC) and NcoI (CCATGG) sites were added to the end of NOS2. This fusion was created and cloned in pUC57 by GenScript. The lyophilized construct was reconstituted and transformed into TOP10 Escherichia coli for amplification. Cells were lysed, and amplified DNA was digested and gel purified. Total DNA was quantitated to calculate copy numbers appropriate for the qRT-PCR standard curve.

### RNA isolation and qRT-PCR.

Whole genital tracts were placed into M-tubes (Miltenyi Biotec) containing 0.5 ml to 1 ml TRIzol (Invitrogen) per 50 mg to 100 mg of tissue. Samples were homogenized with a gentleMACS Dissociator (Miltenyi Biotec) using the RNA_01 program. RNA was isolated from the samples using a RNeasy Midi kit (Qiagen). cDNA was obtained with aSuperScript III first-strand synthesis system using random hexamers (Invitrogen). A 1-μg volume of total RNA was used for qRT-PCR on a StepOnePlus real-time system (Applied Biosystems). Samples were tested in triplicate and amplified using TaqMan primers and probes and TaqMan Advanced Fast master mix according to the instructions of the manufacturer (Applied Biosystems). For absolute transcript number calculations, RNA levels were determined by standard curve. For comparisons using the threshold cycle (2^−ΔΔCT^) method, fold change of infected to uninfected mice was determined after normalization to the HPRT1 housekeeping gene (Applied Biosystems).

### Statistical analysis.

Differences in IFU (infection) levels between treatments and strains of mice were determined using two-way analysis of variance (ANOVA) with the Bonferroni posttest. One-way ANOVA was used with the Bonferroni posttest to determine differences in antibody titers and qRT-PCR absolute transcript numbers. Student's unpaired *t* test was used to determine differences in qRT-PCR fold change analysis between wild-type and anti-CD4-treated mice within the individual time points tested. Statistical calculations were done using Prism 6.0f.

## RESULTS

### Effect of CD4^+^ T cell depletion on the cellular composition of genital tract tissue.

Previous studies have shown that antibody-mediated protection against genital chlamydia infection is dependent on activation and/or recruitment of an effector cell population to the local genital tract tissues by CD4^+^ T cells. Once CD4^+^ T cells have primed the genital tract tissues, they can be eliminated, as they are no longer necessary for the immune protection provided by antibody (12). To determine if the role of CD4^+^ T cells in antibody-mediated protection was to recruit an effector cell population, we assessed whether immune cell emigration to the genital tract was affected by the depletion of CD4^+^ T cells.

**FIG 1:**
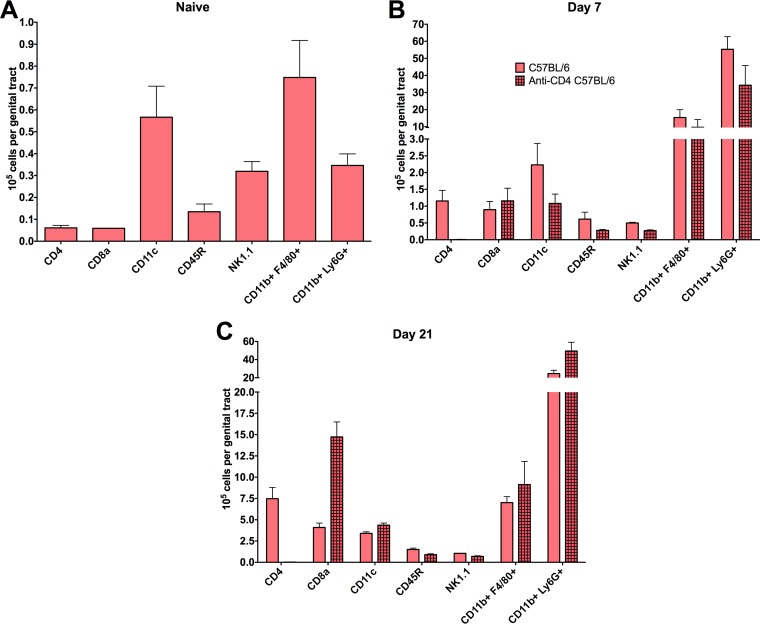
Characterization of genital tract immune cellular infiltrate during primary C. muridarum infection. Whole genital tracts were collected from CD4-depleted and nondepleted naive C57BL/6 mice (A) and at 7 days (B) and 21 days (C) following intravaginal infection. Viable cells were isolated for flow cytometry and stained with antibodies representing the following immune populations (as detailed in Materials and Methods): CD4^+^ T cells (CD4^+^), CD8^+^ T cells (CD8^+^), dendritic cells (CD11c^+^), B cells (CD45R^+^), NK cells (NK1.1^+^), macrophages (CD11b^+^ F4/80^+^), and neutrophils (CD11b^+^ Ly6G^+^). Data represent the mean number of positive-staining cells per genital tract ± standard deviations (SD) from 9 mice/group.

Whole genital tracts from naive C57BL/6 mice and from CD4-depleted and nondepleted mice were harvested at 7 and 21 days following primary infection and processed for flow cytometry analysis (Fig. 1). These time points following primary infection were chosen because CD4^+^ T cell priming of the genital tract tissues occurs during primary infection. Early in the course of primary infection (day 7), comparable levels of CD8^+^ T cells, dendritic cells, B cells, NK cells, macrophages, and neutrophils were present in CD4-depleted and nondepleted mice (Fig. 1B). By day 21 of infection, comparable levels of all cell types were found, with the exception of a larger number of CD8^+^ T cells in depleted mice (Fig. 1C), which are inconsequential in protective immunity to chlamydial genital infection (3, 5, 12). Overall, all immune cell populations were overwhelmingly similar regardless of CD4^+^ T cell depletion. Additionally, Fc receptor (FcR)-bearing cell populations (e.g., neutrophils, macrophages, NK cells, dendritic cells), presumably those that could function with antibody in protective immunity, were unaffected by CD4 depletion. Therefore, CD4^+^ T cell priming of the genital tract for antibody-mediated protective immunity against C. muridarum appears to be unrelated to the recruitment of immune cell populations.

### IFN-γ is required for optimal antibody-mediated protection against infection.

**FIG 2:**
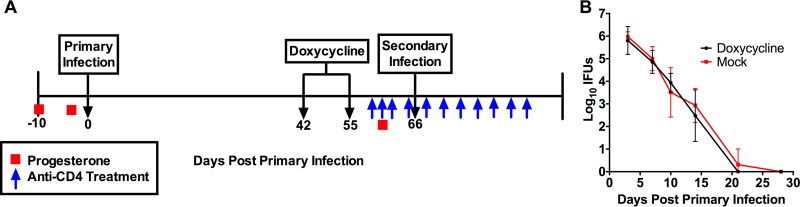
Experimental design for treatment regimens and primary and secondary C. muridarum infection. (A) Mice were treated subcutaneously with 2.5 mg of medroxyprogesterone acetate (Depo-provera) at days −10 and −3 for primary infection and at day −5 for secondary infection and then challenged intravaginally with 5 × 10^4^ inclusion-forming units (IFUs) of C. muridarum. Doxycycline was administered intraperitoneally (i.p.) every day for 2 weeks beginning at day 42 post-primary infection. Mice were rested for 10 days after the last dose of doxycycline, prior to secondary rechallenge. Anti-CD4 antibody was administered i.p. to half the mice of each strain beginning on day −6, day −5, and day −4 prior to secondary rechallenge. Anti-CD4 injections were administered every 3 days until day 20 post-secondary infection. (B) Naive C57BL/6 mice were injected i.p. with doxycycline as described for panel A for a period of 2 weeks and allowed to rest for 10 days. Mice were then infected with 5 × 10^4^ IFU C. muridarum intravaginally, and bacterial loads were determined (mean IFU ± SD; *n* = 5 mice).

Since the recruitment of possible effector cells for antibody-mediated immunity was not impacted by CD4^+^ T cell depletion, we next assessed if CD4^+^ T cells were responsible for the activation of an effector cell population that functioned with antibody to resolve infection. Specifically, we chose to investigate the role of IFN-γ, the highly activating CD4^+^ T cell product. To begin to assess the contribution of IFN-γ to antibody-mediated immunity, we utilized IFN-γ^−/−^ mice, along with C57BL/6 and μMT (antibody-deficient) control mice. The experimental design is schematically depicted in Fig. 2A and is based upon our previous experiments showing the protective contribution of antibody to chlamydial reinfection. One experimental difference from our previous studies, however, is that mice were treated with doxycycline following primary infection to ensure infection resolution before rechallenge. Antibiotic treatment is necessary because IFN-γ^−/−^ mice are unable to completely resolve chlamydial infection (4, 17). To confirm that the treatment/rest regimen did not have any residual effects that could alter the course of infection, naive C57BL/6 mice were treated with doxycycline every day for 2 weeks as described in Materials and Methods and were then rested for 10 days before primary infection. Doxycycline-treated mice showed no residual inhibitory effect of the antibiotic on the course of primary infection (Fig. 2B). Therefore, the treatment/rest regimen did not affect assessment of protective immunity in antibiotic-treated mice.

**FIG 3:**
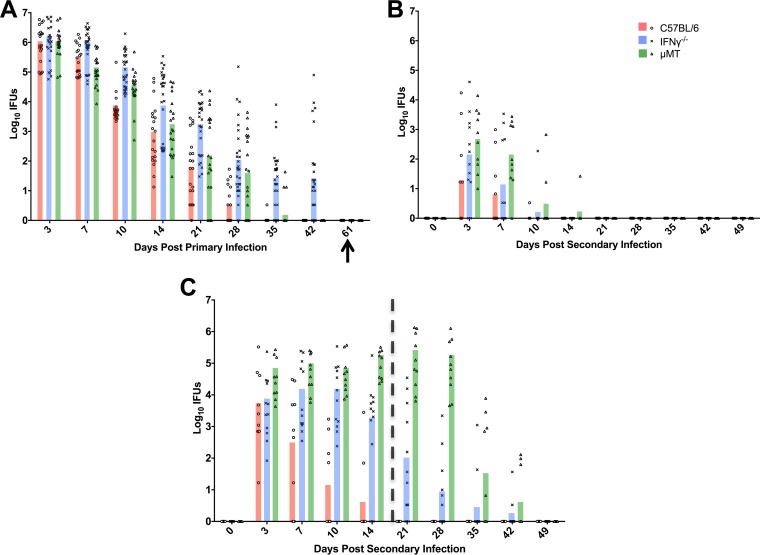
Effect of IFN-γ on bacterial clearance during primary and secondary C. muridarum infection. Shedding of chlamydiae was determined by quantitating IFUs from cervicovaginal swabs collected at various times following infection. (A) Infection course of primary infection in C57BL/6 mice (*n* = 20), IFN-γ^−/−^ mice (*n* = 32), and μMT mice (*n* = 20). The arrow indicates a negative cervicovaginal culture following the conclusion of antibiotic treatment. Half of the mice of each strain (among those described for panel A) were depleted of CD4^+^ T cells as described in Fig. 1, and all mice (including the CD4-depleted and nondepleted mice) were rechallenged with C. muridarum 66 days following primary infection. (B and C) Course of secondary rechallenge infection in nondepleted (B) and CD4-depleted (C) mice. The vertical dashed line at day 20 in panel C represents the last dose of anti-CD4 treatment. Data are presented as IFUs from two independent experiments. Statistical comparisons are listed below rather than on the figures for clarity. For primary infection, statistical significance was determined as follows: for C57BL/6 versus IFN-γ^−/−^, *P* < 0.0001 for days 10 through 42; for IFN-γ^−/−^ versus μMT, *P* < 0.05 for day 7, *P* < 0.01 for day 21, and *P* < 0.0001 for days 35 and 42; for C57BL/6 versus μMT, *P* < 0.05 for day 10 and *P* < 0.0001 for day 28. For nondepleted secondary infection, statistical significance was determined as follows: for C57BL/6 versus IFN-γ^−/−^, *P* < 0.01 for day 3; for IFN-γ^−/−^ versus μMT, *P* < 0.001 for day 7; for C57BL/6 versus μMT, *P* < 0.0001 for days 3 and 7. For CD4-depleted secondary infection, statistical significance was determined as follows: for C57BL/6 versus IFN-γ^−/−^, *P* = 0.0001 for day 7 and *P* < 0.0001 for days 10, 14, and 21; for IFN-γ^−/−^ versus μMT, *P* < 0.0001 for days 14, 21, and 28; for C57BL/6 versus μMT, *P* < 0.05 for day 3, *P* < 0.0001 for days 7 through 28, and *P* < 0.01 for day 35.

To determine the role of IFN-γ in antibody-mediated immunity to genital chlamydia infection, wild-type C57BL/6, μMT, and IFN-γ^−/−^ mice were again used. Primary genital infection had completely resolved in C57BL/6 and μMT mice by 42 days postinfection (Fig. 3A) (5, 11, 12, 18). The resolution of primary infection in IFN-γ^−/−^ mice was remarkably similar to that in C57BL/6 mice and μMT mice for the first few weeks, but IFN-γ^−/−^ mice were unable to completely resolve genital infection (Fig. 3A) (4, 17). However, all strains of mice were culture negative following doxycycline treatment and prior to the initiation of secondary reinfection studies.

To assess efficacy of the antibody response specifically, mice were depleted of CD4^+^ cells and rechallenged with C. muridarum. All strains sufficient in CD4^+^ T cells were markedly protected against reinfection (Fig. 3B) (17). Similarly, wild-type mice depleted of CD4^+^ T cells were also protected due a functional antibody response (Fig. 3C). The severely immunocompromised CD4-depleted μMT mice were unable to resolve infection (5, 12) (Fig. 3C). Secondary infection resolved in CD4-depleted μMT mice only after anti-CD4 treatment had ceased and CD4^+^ T cells repopulated in each animal. Interestingly, CD4-depleted IFN-γ^−/−^ mice displayed an intermediate level of protective immunity to reinfection, characterized by the shedding of large numbers of infectious chlamydiae and an infection course of much longer duration compared to those seen with C57BL/6 controls (Fig. 3C). However, infection did begin to resolve during the time CD4^+^ T cells were functionally depleted (i.e., day 21). Together, these data demonstrate that IFN-γ plays a key role for optimal antibody-mediated immunity to Chlamydia genital reinfection, although other factors may also contribute.

### Chlamydia-specific antibody responses in IFN-γ^−/−^ mice.

**FIG 4:**
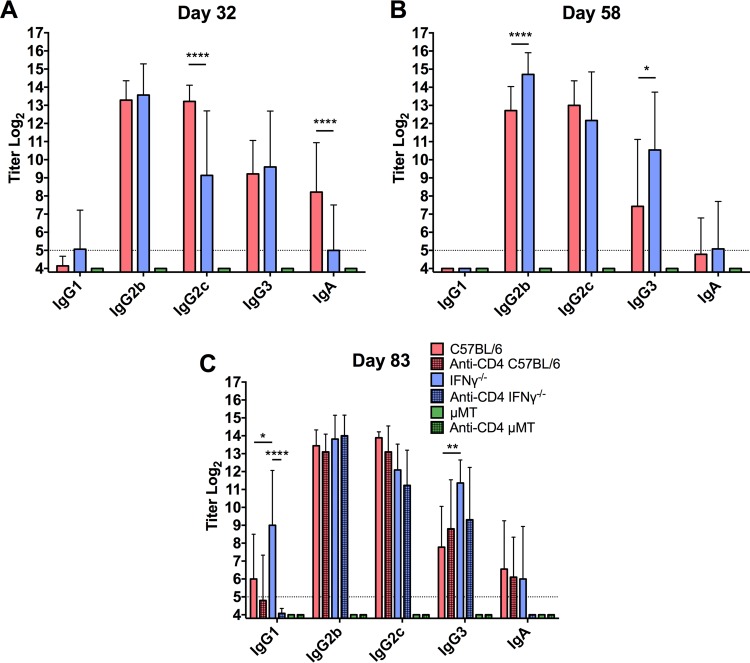
Class- and subclass-specific anti-chlamydial antibody titers during primary and secondary infection. Serum was collected during primary infection (A), before secondary rechallenge (B), and during secondary rechallenge (C) from CD4-depleted and nondepleted C57BL/6, IFN-γ^−/−^, and μMT mice, and chlamydia-specific ELISAs were performed. Indicated days represent times post-primary infectious challenge. μMT mice produced no detectable levels of chlamydia-specific antibodies at any time point. Titers of sera from individual mice were determined, and data are presented as means ± SD for 20 mice/group (C57BL/6 and μMT) or 32 mice/group (IFN-γ^−/−^). *, *P* < 0.05; **, *P* < 0.01; ***, *P* < 0.001; ****, *P* < 0.0001.

IFN-γ is known to play a role in antibody production and isotype switching in response to an infection (19–22), suggesting that the diminished antibody-mediated protection observed during secondary infection in CD4-depleted IFN-γ^−/−^ mice could be due to a different, less protective antibody response in the absence of IFN-γ. To determine if the antibody response of IFN-γ^−/−^ mice differed from the antibody response of wild-type C57BL/6 mice, serum was collected during primary infection, before CD4^+^ T cell depletion, and during secondary infection. ELISA analysis determined that, while the IgG2c response was somewhat delayed during primary infection in the IFN-γ^−/−^ mice, C57BL/6 and IFN-γ^−/−^ mice had comparable immunoglobulin class and subclass chlamydia-specific antibody titers prior to secondary infection (Fig. 4A and B). Serum antibody titers during secondary infection confirmed that these responses were maintained during infection and that CD4^+^ T cell depletion prior to reinfection had little to no effect on the magnitude of the antibody response (Fig. 4C). Therefore, the decrease in protection seen in CD4-depleted IFN-γ^−/−^ mice was not due to differences in the chlamydia-specific antibody response.

### Immune cell genital tract infiltration following infection in CD4-depleted IFN-γ^−/−^ mice.

**FIG 5:**
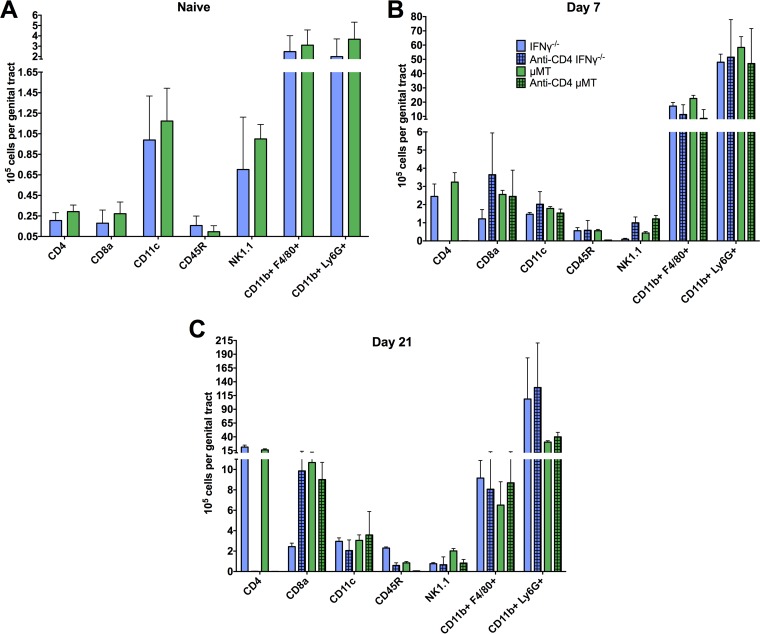
Immune cell infiltration of the genital tract during C. muridarum primary infection in CD4-depleted and nondepleted IFN-γ^−/−^ and μMT mice. Whole genital tracts were collected, prepared, and stained as described for Fig. 1 in depleted and nondepleted naive IFN-γ^−/−^ and μMT mice (A) and at 7 days (B) and 21 days (C) following primary intravaginal infection. Data represent the mean number of positive-staining cells per genital tract ± SD of nondepleted IFN-γ^−/−^ (*n* = 9) and μMT (*n* = 9) mice and of depleted IFN-γ^−/−^ (*n* = 6) and μMT (*n* = 6) mice, respectively.

Although immune populations emigrate normally to the genital tract following CD4^+^ T cell depletion in C57BL/6 mice (Fig. 1), IFN-γ has been shown to recruit neutrophils and other cell populations to sites of infection (23). In order to rule out the possibility of a defect in IFN-γ-mediated recruitment of an effector population in CD4-depleted IFN-γ^−/−^ mice, IFN-γ^−/−^ and μMT control mice were depleted of CD4^+^ T cells during primary infection (as shown in Fig. 1) and levels of immune cell populations present in the genital tract were determined by flow cytometry. Overall, CD4^+^ T cell depletion did not affect the recruitment of major immune populations to the genital tract at any time point during primary chlamydial infection in IFN-γ^−/−^ or μMT mice (Fig. 5). Thus, the diminished protection seen in CD4-depleted IFN-γ^−/−^ mice was not due to a defect in CD4-mediated recruitment of an effector population.

### Effect of CD4^+^ T cell depletion on the global activation status of infected genital tracts.

**FIG 6:**
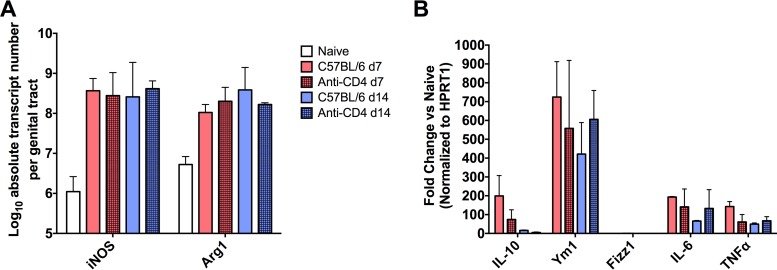
Effect of CD4^+^ T cell depletion on the activation state of the murine genital tract during primary C. muridarum infection. RNA was harvested from whole genital tracts from CD4-depleted or nondepleted C57BL/6 mice (*n* = 4) at the noted time points during primary infection for analysis by quantitative reverse transcriptase PCR. Expression of activation-related mRNAs was determined. (A) Inducible nitric oxide synthase (NOS2) and arginase 1 (Arg1) total transcript numbers per genital tract in naive, CD4-depleted, and nondepleted mice were calculated. (B) mRNA levels of IL-10, Ym1, Fizz1, IL-6, and TNF-α in CD4-depleted and nondepleted C57BL/6 mice relative to naive controls were determined. Data were normalized to the HPRT1 housekeeping gene. Numbers represent means ± SD (*n* = 4).

IFN-γ is a potent activator of many cellular immune processes in response to infection. The necessity of IFN-γ for optimal antibody-mediated protection against reinfection led us to ask if we could establish global differences in the immune activation status of the genital tract during C. muridarum infection in the presence and absence of CD4^+^ T cells. Specifically, since macrophages and neutrophils are effector cells that interact with antibody and can be activated by IFN-γ, we evaluated the effect of CD4^+^ T cell depletion on various macrophage and neutrophil activation markers. To determine the type and magnitude of immune activation in the genital tract, we measured mRNA levels of downstream products of IFN-γ activation, as well as other markers representing a spectrum of inflammation states, including inducible nitric oxide synthase, arginase I (Arg1), Ym1, Fizz1, interleukin 10 (IL-10), and interleukin 6 (IL-6). To determine the specific effect of CD4^+^ T cells on activation, C57BL/6 mice were depleted of CD4^+^ T cells during primary C. muridarum infection and whole genital tract qRT-PCR was performed on days 7 and 14, comparing genital tract mRNA levels in CD4-depleted mice to the levels in the non-CD4-depleted controls. iNOS transcript levels were greatly increased over the levels seen with naive mice during infection as expected, but similar transcript numbers were seen in CD4-depleted and nondepleted mice at days 7 and 14 (Fig. 6A). Similar levels of IL-10, Ym1, Fizz1, IL-6, and tumor necrosis factor alpha (TNF-α) were also observed regardless of CD4^+^ T cell depletion status (Fig. 6B).

## DISCUSSION

There is a great need for a vaccine to aid in prevention of chlamydial infection on a global scale. While previous studies have shown that both CD4^+^ T cells (2–5) and antibody (5, 11, 12) play central roles in protection against C. muridarum infection, we sought in this study to uncover the mechanism(s) by which antibody mediates immunity. Delineation of specific mechanisms of protection will greatly facilitate the development of an efficacious vaccine. Previous studies have shown that antibody provides a striking level of protective immunity to chlamydial genital infection (5, 11, 12) and that antibody-mediated protection is dependent on CD4^+^ T cells orchestrating the recruitment and/or activation of an effector cell population (12), since antibody does not appear to protect *in vivo* via direct chlamydial neutralization (12, 24). While IFN-γ has been shown to be directly and indirectly involved in the recruitment of neutrophils and inflammatory macrophages (two major FcR-bearing populations present during chlamydial infection) in a variety of infection settings (25–28), we showed in the current study that immune cell recruitment is not negatively impacted by the absence of IFN-γ during C. muridarum genital infection. Importantly, several additional cytokines and chemokines secreted by CD4^+^ T cells, including IL-17 and IL-6, are proinflammatory and capable of recruiting immune cells to the genital tract during infection (29, 30). We found that, similarly to IFN-γ^−/−^ mice, CD4 depletion had minimal impact on overall immune cell infiltration during infection in all mouse strains tested, with the exception of an increase in CD8^+^ T cell levels at day 21 postinfection. However, this increase in CD8^+^ T cell levels does not equate with protection, as CD4^+^ T cell-depleted CD8^+^ T cell-sufficient mice are incapable of resolving C. muridarum infection or priming the genital tract for antibody-mediated immunity (3, 5, 12). The similarities in cellular infiltration between CD4-depleted and nondepleted mice strongly support the notion that IFN-γ-mediated effector cell activation, rather than effector cell recruitment, is central to optimal antibody-mediated immunity.

It is generally accepted that there are both IFN-γ-dependent and -independent mechanisms that aid in the clearance of primary genital C. muridarum infection (4, 17). IFN-γ is secreted in large quantities by CD4^+^ T cells during chlamydial infection and is known to play a role in immunoglobulin class switching, B cell maturation, and activation of several immune cell populations and processes during bacterial infections (19, 31–37). In our current study, however, we identify a new role for IFN-γ in the protective antibody response against C. muridarum reinfection. During primary and secondary infections, the chlamydia-specific antibody responses were largely similar in magnitude and class/subclass specificity in C57BL/6 and IFN-γ^−/−^ mice, suggesting that the prominent role for IFN-γ is independent of class switching (Fig. 4) (35). Moreover, it was expected on the basis of previous studies (4, 17) that IFN-γ would play an important role in the cellular T_H_1 response to genital chlamydia infection, but its necessity for antibody-mediated immunity (not antibody production) in chlamydial genital infection shows an additional functional role of this cytokine in chlamydial immunity.

IFN-γ was required for optimal antibody-mediated protection. Infection resolution began prior to the end of CD4^+^ T cell depletion in IFN-γ^−/−^ mice (Fig. 3C), indicating that other mediators must be involved in addition to IFN-γ. CD4^+^ T cells secrete a plethora of activating cytokines during C. muridarum infection, including IL-17 (38, 39). IL-17 plays multiple roles, including recruiting and activating various immune cell populations at the local infection site, during infections by bacterial species such as Bordetella pertussis and Klebsiella pneumoniae (40–43). Furthermore, IL-17 is capable of acting both alone and together with IFN-γ in reducing chlamydial burden in epithelial cells and macrophages *in vitro* (44) and has also been shown to act synergistically with IFN-γ to kill pathogens *in vivo* (45–47). Therefore, because genital C. muridarum infection induces a T_H_17 response and IL-17 activates phagocytic cells (38, 39, 44, 48, 49), it will be important to further evaluate the contribution of IL-17 in antibody-mediated chlamydial immunity.

The direct mechanism(s) of antibody-mediated protection has not been fully elucidated. However, previous work has suggested a variety of roles for antibody during chlamydial infection, including both indirect immune support such as enhancement of T cell activation by concentrating antigen within professional antigen-presenting cells (APCs) and facilitation of epithelial cell killing via antibody-dependent cell-mediated cytotoxicity (ADCC) by macrophages (50, 51). It has also been proposed that antibody plays a key role in preventing the dissemination of chlamydia to extragenital sites (52), which may or may not be related to the dissemination seen in IFN-γ^−/−^ mice (4, 17). While our current study did not uncover the specific IFN-γ-mediated activation event(s) required for antibody to protect during genital chlamydia infection, our data allude to an important role for the highly activating IFN-γ in enhancing the antimicrobial activity of phagocytes and/or increasing the expression of FcRs to augment phagocytosis of antibody-coated chlamydiae, as has been shown for other bacteria (53–56). Further supporting this notion is the dramatic increase in the number of macrophages and neutrophils (both FcR-bearing phagocytic populations) in the genital tissue following infection with C. muridarum (Fig. 1 and 5). Additionally, for another intracellular bacterium, Francisella tularensis, phagocyte-dependent antibody-mediated protection is completely dependent upon the secretion of IFN-γ by CD4^+^ T cells (57). The concept of increased phagocyte activation and killing of antibody-opsonized intracellular pathogens is not new (58–62), but in the current study we have uncovered a possible unique mechanism by which the genital tract tissues are primed during primary chlamydial infection for subsequent protection to occur via phagocyte-antibody interactions. While we hypothesize that increased phagocytic killing is largely responsible for antibody-mediated protection, combinations of multiple mechanisms and mediators are likely required for complete immunity. The lack of differences seen in the global activation state of the infected genital tract with CD4^+^ T cell depletion as measured by qRT-PCR (Fig. 6) may be due to other immune effectors playing compensatory roles to regulate the transcripts measured.

A direct role for antibody in mediating immunity against human genital chlamydia infection has not been firmly established, even though subjects with ongoing C. trachomatis genital infection produce significant chlamydia-specific antibody responses (63). While one study found an association between high IgA titers and bacterial clearance in the endocervix (64), most chlamydia serologic responses do not correlate with protective immunity. This lack of a strong correlation between antibodies and protective immunity in humans is not unlike what was initially concluded from the murine model of C. muridarum genital infection. Specifically, mice develop striking protective immunity in the absence of antibody (18), which led to the notion that antibody was unimportant in chlamydial immunity. The considerable protective role of antibody in chlamydial immunity was not appreciated until the protective CD4^+^ T cell responses were eliminated (5, 11, 12). Thus, perhaps the absence of obvious antibody-mediated protection in humans results from the inability to clearly delineate protective CD4^+^ T cell responses and protective antibody responses. An alternative explanation for the absence of a strong correlation between antibody and protective immunity to human chlamydial genital infection comes from our previous work using the murine infection model (5, 11, 12, 14) and this current study. We have shown that antibody protects only following CD4^+^ T cell priming of the genital tract tissues. Because the majority of human chlamydial genital infections are without signs or symptoms, suggesting mild inflammation, perhaps this mild inflammatory response results in incomplete or ineffective priming of the genital tract by CD4 T^+^ cells during infection. Further studies will be needed to demonstrate whether equivalent immune mechanisms function to resolve human infection and to protect against reinfection.

Overall, our data provide important insight into the ability of antibody to protect after CD4^+^ T cell priming of the genital tract during initial Chlamydia infection. We provide evidence that IFN-γ is important for genital tract priming and that activation of an effector cell population(s) by IFN-γ may be key to this response. Further elucidation of the mechanisms of protective adaptive immune responses, both cellular and humoral, during chlamydia infection will provide further insight into the type(s) of response(s) that needs to be elicited by a successful vaccine.
